# Dr. A. E. Willard

**Published:** 1898-12

**Authors:** 


Dr. A. E. Willard, of Friendship, Allegany county, N. Y., died at his home in that village, November 20, 1898, aged 67 years. He was one of the most respected physicians in the Southern tier, and had conducted a successful practice since his graduation at the Buffalo

University in 1864. His father, Dr. E. H. Willard, settled in Friendship in 1841 and practised medicine until his death in 1886. He represented Allegany county in the Assembly in 1849.

Dr. A. E. Willard had been secretary of the Medical Society of Allegany county for fifteen years, and was withal a prominent citizen of that region. He is survived by a widow and two daughters. His funeral, held November 22d, was largely attended by the most prominent residents of the vicinity.

